# Comparison of Oral, Intranasal and Aerosol Administration of Amiodarone in Rats as a Model of Pulmonary Phospholipidosis

**DOI:** 10.3390/pharmaceutics11070345

**Published:** 2019-07-17

**Authors:** Aateka Patel, Ewelina Hoffman, Doug Ball, Jan Klapwijk, Rory T. Steven, Alex Dexter, Josephine Bunch, Daniel Baker, Darragh Murnane, Victoria Hutter, Clive Page, Lea Ann Dailey, Ben Forbes

**Affiliations:** 1 Sackler Institute of Pulmonary Pharmacology, Faculty of Life Sciences & Medicine, Franklin-Wilkins Building, King’s College London, 150 Stamford Street, London SE1 9NH, UK; 2 Institute of Pharmaceutical Science, King’s College London, Franklin-Wilkins Building, King’s College London, 150 Stamford Street, London SE1 9NH, UK; 3 Centre for Topical Drug Delivery and Toxicology, School of Life and Medical Sciences, University of Hertfordshire, Hatfield, Herts AL10 9AB, UK; 4 Department of Pharmaceutical Biochemistry and Molecular Diagnostics, Pharmacy Faculty, Medical University of Lodz, 90-151 Lodz, Poland; 5 Allergic Inflammation Discovery Performance Unit, GlaxoSmithKline, Gunnelswood Road, Stevenage, Herts SG1 2NY, UK; 6 Translational Medicine and Comparative Pathobiology, GlaxoSmithKline, Park Road, Ware, Hertfordshire SG12 0DP, UK; 7 National Physical Laboratory, Teddington, London TW11 0LW, UK; 8 Institute of Pharmaceutical Technology and Biopharmacy, Martin Luther University Halle-Wittenberg, Wolfgang-Langenbeck-Str. 4, 06108 Halle (Saale), Germany

**Keywords:** phospholipidosis, amiodarone, foamy alveolar macrophages, di-22:6 bis-monoacylglycerol, mass spectrometry imaging, high content analysis

## Abstract

‘Foamy’ alveolar macrophages (FAM) observed in nonclinical toxicology studies during inhaled drug development may indicate drug-induced phospholipidosis, but can also derive from adaptive non-adverse mechanisms. Orally administered amiodarone is currently used as a model of pulmonary phospholipidosis and it was hypothesized that aerosol administration would produce phospholipidosis-induced FAM that could be characterized and used in comparative inhalation toxicology. Han-Wistar rats were given amiodarone via (1) intranasal administration (6.25 mg/kg) on two days, (2) aerosol administration (3 mg/kg) on two days, (3) aerosol administration (10 mg/kg) followed by three days of 30 mg/kg or (4) oral administration (100 mg/kg) for 7 days. Alveolar macrophages in bronchoalveolar lavage were evaluated by differential cell counting and high content fluorescence imaging. Histopathology and mass-spectrometry imaging (MSI) were performed on lung slices. The higher dose aerosolised amiodarone caused transient pulmonary inflammation (*p* < 0.05), but only oral amiodarone resulted in FAM (*p* < 0.001). MSI of the lungs of orally treated rats revealed a homogenous distribution of amiodarone and a putative phospholipidosis marker, di-22:6 bis-monoacylglycerol, throughout lung tissue whereas aerosol administration resulted in localization of both compounds around the airway lumen. Thus, unlike oral administration, aerosolised amiodarone failed to produce the expected FAM responses.

## 1. Introduction

Inhaled therapies for asthma were first developed in the 1950s and remain the preferred route of administration for treating the disease [1]. However, there have been few new classes of drugs licensed during the past 50 years, with many that perform well in pre-clinical animal studies failing in pre-clinical development owing to a lack of safety and/or efficacy in humans [2]. Failure to translate promising drug effects from pre-clinical assays and animal studies to humans has led to questions regarding the relevance of current pre-clinical models, and a demand for more predictive in vivo and in vitro tools.

One of the difficulties in non-clinical assessment of inhaled drug safety is uncertainties around the alveolar macrophage (AM) response in animals and its relevance to safety in the clinic [2]. A typical finding in toxicity studies with inhaled formulations of new drugs is the induction of foamy alveolar macrophages (FAM) which may or may not be indicative of subsequent adverse events. Despite not knowing the longer term effects of inducing FAM, the presence of such cells in the lung in toxicology experiments can stop the further development of inhaled drug candidates. “Foamy macrophage” is a term used to describe the vacuolated, enlarged appearance of an AM, observable by light microscopy, due to the presence of lamellar bodies, or an accumulation of lipids or drug particles in the cytoplasm [2,3]. It is often unclear whether macrophage responses are due to general particulate overload [4] or pharmacologically-driven [2,5] and it is thought that different pathways underpin different mechanisms. The foamy appearance may also be accompanied by increase in macrophage numbers and activation. Any of these effects may represent an adaptive response to the administered material or the initial stages of an adverse effect.

The inability to discriminate between adaptive and adverse responses triggers additional in vivo assessments to determine whether there are secondary consequences of FAM appearance, such as inflammation, and to make go/no-go decisions on candidate drugs progressing to clinic phases of development which can lead to drugs exhibiting this effect being discontinued, perhaps prematurely, from further development. To improve inhaled drug development, a better understanding is required of FAM biology and the significance of this finding when observed in species routinely used to predict safety of inhaled medicines to be used in humans. This knowledge would hopefully lead to better lead compound selection and a reduction in the costly and repetitive toxicological studies in animals required to optimise safe doses of inhaled medicines.

Previous work in our laboratory has focused on the design of an in vitro high content “foamy macrophage” assay platform [4] that may be used in the early development stages to screen for problematic compounds or formulations with the aim of excluding them from the development pathway prior to animal studies. Based on automated fluorescence imaging, the high content analysis (HCA) assay provides quantitative data on morphometric parameters and vacuolation patterns, and can be combined with a variety of markers for phenotypic and functional characterisation of macrophages. Amiodarone has been used as a positive control to induce a FAM appearance in the development of the in vitro screening platform and was therefore selected as a model compound to investigate how well the in vitro assay outputs correlate with macrophages lavaged from an in vivo model following treatment with this drug when administered by inhalation.

Amiodarone is a prototypic cationic amphiphilic drug (CAD), well-known for its pulmonary toxicity profile and ability to induce phospholipidosis in the AM population [6,7,8]. Oral administration of amiodarone is associated with serious pulmonary toxicity, including acute or subacute pneumonitis [8,9], alveolitis [10], pulmonary infiltrates, pleural disease, and the abnormal accumulation of phospholipids in cells and tissues [6,8,10,11,12], without resulting in fibrosis [12,13]. As well as the inflammatory effects, there is significant evidence of macrophages developing a foamy phenotype [7,14,15,16,17]. When administered intratracheally (i.t.) amiodarone causes pulmonary fibrosis [18,19,20] as well as an increase in the number of inflammatory cells (macrophages, neutrophils and eosinophils) recovered from bronchoalveolar lavage (BAL) [12,18,19,21].

In this study, it was hypothesised that administration of amiodarone as an inhaled dry powder would provide a more evenly distributed, physically and quantitatively relevant exposure scenario compared to intranasal (i.n.), i.t. instillation or oral administration, and therefore provide an appropriate model and positive control for studying the FAM response to inhaled medicines. The effects of inhaled amiodarone powder aerosols have not been reported previously. Therefore, the aim of this study was to characterise the effects of an inhaled powder formulation of amiodarone in the rat, a species commonly used in regulatory toxicology experiments. Prior to the main aerosol study, a small number of animals were dosed intranasally with 6.25 mg/kg amiodarone (on two alternate days) to evaluate dose tolerability in Wistar Han rats, since previous reports in the literature on i.t. instillation of amiodarone (also 6.25 mg/kg) to F344 rats described rather severe toxicological effects ranging from inflammation to pulmonary fibrosis [18,19]. As a further comparison, an oral dosing study (100 mg/kg over seven consecutive days) was performed, such that the results from all administration routes could be compared within the same study. We evaluated BAL fluid inflammatory markers and used the HCA assay platform to characterise the BAL macrophage population, as well as conventional histopathology and mass-spectrometry imaging (MSI) of lung tissue slices. Results of the HCA analysis were compared with data from a previous in vitro investigation of amiodarone exposure to a rat macrophage cell line and primary rat cells from naïve rat lungs in order to determine whether in vitro HCA results reflect the in vivo response.

## 2. Materials and Methods

### 2.1. Materials

Methanol (LC-MS grade) was purchased from Fisher Scientific (Leicestershire, UK); purified water, ELGA Purelab Option (Marlow, UK); MALDI matrices and analytes, 2,5-dihydroxybenzoic acid (2,5-DHB, >99%), 9-aminoacrinid (9AA, ≥99.5%), d-(+)-raffinose pentahydrate (≥98%) were purchased from Sigma-Aldrich (Dorset, UK) and used as supplied. Amiodarone hydrochloride (≥98%) was purchased from Sigma-Aldrich (Dorset, UK) and used as supplied or blended with lactose monohydrate, Lactohale (GMS 15284, Grade 4), EP and USP and JP, bovine (DFE Pharma, Goch, Germany) for use by inhalation. All other materials were of analytical grade.

### 2.2. Amiodarone Characterisation

#### 2.2.1. Aerodynamic Particle Sizer

Aerosols were monitored using a TSI aerodynamic particle sizer (APS) during pre-study aerosol generation trials and during inhalation exposure of animals; data from the APS was used to further validate this system, but was not used to report the achieved dose or particle size. The APS was sampled at a calibrated flow rate of 0.2 L/min for a period of 30 s every minute. The APS data was used to confirm that particle number and size were consistent with expected values determined from pre-study aerosol generation trials.

#### 2.2.2. Chemical Analysis of Amiodarone and Target Dose

Amiodarone was micronised to give a mass median aerodynamic diameter of 2.1 µm with a geometric standard deviation of 2.1, indicating that the particle size was consistent and within the respirable range of 1–3 µm for rats. The amiodarone concentration in each exposure chamber was determined in duplicate during inhalation exposure of animals. Samples were taken from a sampling port by drawing a measured volume of air through a 37 mm GF-A glass fibre filter at a rate of 2.0 L/min for 1 min fitted to an open face sampler attached to the chamber sampling port. The filters were weighed before and after sampling for gravimetric analysis of total particulate concentration. For particle size measurements a measured volume of air was drawn through a Marple personal cascade impactor (model 296) attached to a sampling pump from the exposure chamber at a rate of 2.0 L/min for 1 min. All concentrations and particle size samples were analysed within the Safety Assessment Dispensary Group at GlaxoSmithKline, Ware, UK. The target dose for protocol one and protocol two was calculated using the Alexander equation [22]:

(1) $$\mathrm{DD}=\frac{\left(\mathrm{CC}\times \mathrm{RMV}\times T\right)}{\mathrm{BW}}.$$

The RMV for rats was calculated according to the formula:

(2) $$\mathrm{RMV}\;\left(L/\min\right)=0.608\times \mathrm{BW}\;{\left(\mathrm{kg}\right)}^{0.852},$$

where: DD (mg/kg) = Delivered Dose (whole body dose); CC (mg/L) = Cloud Concentration = amount on filter (0.575 mg)/filter sample volume (4 L) (filter sample volume = filter sample rate (2 L/min) × filter sample time (2 min)); RMV (L/min) = Respiratory Minute Volume (0.25 L/min); T (min) = Time of Exposure; BW (kg) = Body Weight (average weight of rats during a dose run).

### 2.3. Animals

All experiments were approved by the Home Office and conducted in accordance with the Animals (Scientific Procedures) Act 1986. Protocols were approved by the local ethics committee of King’s College London and by the GSK animal welfare and ethical review committee (AWERB), 18 February 2016, project identification code: N37752-1/SP1600088. In vivo experiments were performed with male Wistar Han rats supplied by Charles River, 7–9 weeks of age (approximately 250–300 g).

### 2.4. Dosing of Amiodarone

#### 2.4.1. Intranasal Administration of Amiodarone

Amiodarone hydrochloride (6.25 mg/mL stock solution) [18] was dissolved in saline at 60–65 °C [20,21] and allowed to cool to room temperature. On days −2 and 0 rats were anaesthetised with isoflurane 3–5% *v*/*v* in O_2_ at a flow rate of 1.0 L/min before i.n. administration of amiodarone (6.25 mg/kg) or vehicle, 0.9% physiological saline, to the lungs [18,19]. Rats were observed until consciousness was regained, then returned to their home cages and holding room (unless euthanised).

#### 2.4.2. Aerosol Administration of Amiodarone (Dosing Protocol One)

All rats were placed in restraint cones and attached randomly to one of the 24 ports on the aerosolised dust generation (ADG) tower. On days −2 and 0, control rats were exposed to air for 30 min using the capsule-based aerosol generator (CBAG) mechanism under the same conditions for dry powder inhalation. On days −2 and 0, another cohort of rats were exposed for 30 min to a dry powder aerosol of 100% micronised amiodarone, target dose 3 mg/kg using the CBAG mechanism with a baffle in the nozzle section. A total of 204 capsules, each filled with 1 mg of micronised amiodarone, were used for the inhalation dosing session.

#### 2.4.3. Aerosol Administration of Amiodarone (Dosing Protocol Two)

All rats were placed in restraint cones and attached randomly to one of the 24 ports on the ADG tower. Animals were dosed with either air or 15% micronised amiodarone in 85% inhalation grade lactose for 30 min with increasing daily doses of 10 mg/kg (day −3) and 30 mg/kg (days −2, 1 and 0) using a Wright’s dust feeder (WDF).

#### 2.4.4. Oral Administration of Amiodarone

Male Wistar Han rats (Charles River), of slightly larger body weight (300–395 g) were dosed orally by gavage using nelaton catheters (size 8) with amiodarone (hydrochloride salt) 100 mg/kg, suspended in 1% *w*/*v* methylcellulose for seven consecutive days.

### 2.5. Differential Cell Counts of BAL Cellular Fraction

Rats were sacrificed humanely with an overdose of sodium pentobarbitone (400 mg/rat) administered intra-peritoneally on days 1, 7 or 28 post-dosing. Death was confirmed by exsanguination. The lungs were instilled with 3 × 5 mL BAL rinse fluid (3.72 g ethylenediaminetetraacetic acid/1 g bovine serum albumin/L in phosphate buffered saline (PBS)). The total number of cells were counted using an Improved Neubauer haemocytometer. Cytospin preparations were stained with Diff-Quick (Thermo Fisher Scientific, Waltham, MA, USA) for differential cell count quantification. A total of 200 cells were evaluated to determine the proportion of neutrophils, eosinophils and macrophages using standard morphological criteria. The AM population was further assessed by evaluating 100 macrophages to subcategorise their morphology as normal, foamy or coarsely vacuolated.

### 2.6. High Content Analysis of BAL Macrophages

Rat AM recovered from the cellular fraction of the BAL were centrifuged at 250 G for 5 min at 4 °C. Cell pellets were re-suspended in 1 mL of complete culture medium comprising of phenol red free RPMI-1640 medium (Sigma-Aldrich, Dorset, UK) supplemented with 10% *v*/*v* heat inactivated fetal bovine serum (Sigma-Aldrich, Dorset, UK), 2 mM l-glutamine (Sigma-Aldrich, Dorset, UK) and 100 IU/mL penicillin/100 µg/mL streptomycin (Gibco, Life Technologies, Paisley, UK). For experiments, cells were seeded onto bottom clear black 96-well plates (Greiner Bio-One, Gloucester, UK) at a density of 1.5 × 10^4^ cells/well in 100 µL of complete cell culture medium. The plate was centrifuged at 380 g for 5 min at 20 °C and the cells were then incubated for 2 h in a humidified atmosphere at 37 °C with 5% *v*/*v* CO_2_ for macrophage attachment [23] before undergoing the fluorescence staining procedure.

Once the cells had attached to the 96-well plate after 2 h isolation, non-adherent cells were aspirated and 200 µL of fresh phenol red free media was added to all wells containing cells. Cell health and morphology assessment was completed according to an established protocol [24]. In brief, cells were incubated for 30 min with a florescent staining cocktail containing: Hoechst 33342 (10 µg/mL), MitoTracker Red (300 nM) and Image-It Dead Green (25 nM) (Invitrogen, Renfrewshire, UK), followed by washing with 100 μL PBS, and fixed with 4% *w*/*v* paraformaldehyde for 15 min. The cell cytoplasm was with Cell Mask Deep Red (Invitrogen, Renfrewshire, UK) diluted 1:1000 (according to the manufacturer’s protocol) for 100 min in the dark at 25 °C. Cells were then washed once with PBS as described above before imaging.

To quantify lipid content, cells were seeded onto 96-well plates at the density of 1.5 × 10^4^ cells/well, in complete cell culture media containing HCS LipidTox Phospholipid Red (Invitrogen, Renfrewshire, UK) diluted 1:1000 (according to the manufacturer’s protocol). The cells were incubated for 2 h in a humidified atmosphere at 37 °C with 5% *v*/*v* CO_2_. Following incubation cells were washed once with 100 μL PBS and fixed for 20 min with 4% *w*/*v* paraformaldehyde containing Hoechst 33342 (10 µg/mL). Cells were washed once with 100 µL PBS before incubation with HCS LipidTox Green (diluted 1:1000) for 30 min for detection of neutral lipids.

Cells from both assays were imaged using the In Cell Analyser 6000 (GE Healthcare, Little Chalfont, Bucks, UK). Images in standard 2D mode were captured with a 40× objective. Internal controls of untreated cells contained 200 μM carbonyl cyanide-4-(trifluoromethoxy) phenylhydrazone as a mitochondrial activity control and 0.5% *v*/*v* Triton X-100 as a permeability control. Images were analysed using the InCell Developer v 1.9.2 software (GE Healthcare, Little Chalfont, Bucks, UK) [24]. Not all data fitted a normal Gaussian distribution, therefore, median values were calculated and compared to elevate the effect of amiodarone.

### 2.7. Lung Histology

Following lavage, the left and intermediate lung lobes were fixed in 10% neutral buffered formalin. Three transverse sections were cut through the left lung (superior, median and caudal regions) and embedded in paraffin wax blocks. Sections of 3–5 μM were cut and stained with hematoxylin and eosin for visualization of inflammatory cell infiltrate and assessment of histopathological lesions.

### 2.8. Mass Spectrometry Imaging and Analysis

The right lung lobe was excised, weighed and laid on a foil lined mega cassette with the lobe gently spread out. The mega cassette was placed on dry ice to freeze, and then stored in a labelled plastic bag and transferred to an ultra-cold freezer at −70 to −90 °C prior to dispatch. Sample transport was subsequently carried out on dry ice. Samples were sectioned at 14 µm by cryo-microtome (CM 1850, Leica, Milton Keynes, UK) and thaw mounted onto glass microscope slides (Superfrost Plus, Thermo Fisher, Waltham, MA, USA) prior to storage at −80 °C until needed. Samples were placed in a vacuum desiccator for 30 min prior to analysis, returning to room temperature whilst minimizing condensation.

DESI MSI was performed at 50 × 50 or 100 × 100 µm, scan time of 0.48 s and stage speeds of 100 and 200 µm/s respectively. A solvent system of 95:5 methanol:water (*v*:*v*) with 0.001 mg/mL raffinose (lock mass/internal standard) was employed. Quality control and optimization was carried out on rhodamine (from a red Sharpie pen) or *m/z* 666 (from a black Staedtler pen) prior to tissue analysis where an ion intensity of >10^6^ was used as a passing threshold. Additionally, the characteristics of the eroded spot from the DESI spray were assessed on a thin film of rhodamine sublimated onto glass [25]: a tightly focussed, approximately circular region clearly eroding through the entire layer in ≤1 s was used.

Data were converted from proprietary Waters.RAW format into imzML using ProteoWizard [26] and the imzML converter [27], and imported into Matlab (version 2017a and statistics and image processing toolbox, The Math-Works, Inc., Natick, MA, USA) using SpectralAnalysis [28]. Ion images were derived from the area under a given peak at full width half maximum.

### 2.9. Statistics

GraphPad Prism 7.01 software was used for all statistical analyses. Data was analysed for normality using the Shapiro–Wilk test. BAL cell counts from amiodarone and air-challenged rats were compared using a two-way ANOVA with a post-hoc Bonferroni test. Morphology and lipid content data were assessed using a Mann–Whitney test. Statistical significance was evaluated at a 95% confidence level (*p* < 0.05).

## 3. Results

### 3.1. Assessment of Inflammation and Phospholipidosis Markers in the BAL Cellular Fraction

**Figure 1:**
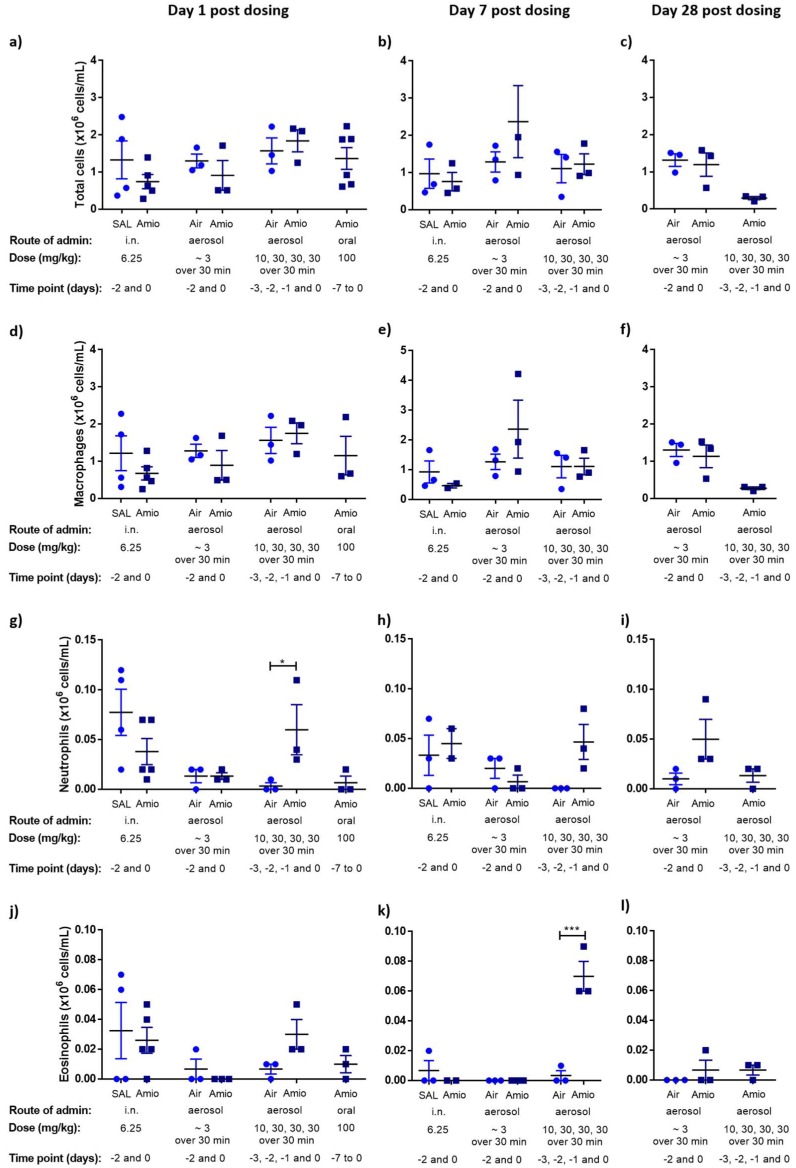
Effect of intranasal (i.n.), aerosol or oral amiodarone exposure on the total number of cells, macrophages, neutrophils and eosinophils recovered by bronchoalveolar lavage (BAL). Rats were exposed to amiodarone, sterile saline (SAL) or air (control) at various time points (days) and subjected to BAL at day 1, day 7 or day 28 post-dosing (**a**–**c**). Total cell number recovered by BAL, (**d**–**f**) number of alveolar macrophages recovered by BAL, differential counts of (**g**–**i**) neutrophils and (**j**–**l**) eosinophils recovered by BAL. Columns represent mean ± SEM (*n* = 2–5 per treatment group); two-way ANOVA with post hoc Bonferroni Test, Difference from respective saline or air controls; * *p* < 0.05, *** *p* < 0.001, statistically significant compared with the respective control, two-way ANOVA with post hoc Bonferroni test.

All dosing protocols included a group of animals (*n* = 3–5 animals) culled one day following the final dose. The i.n. study also included a cull on day 7 post-final dose and both aerosol studies included cull groups on days 7 and 28 post-final dose. All control groups (saline or air treated) showed outcomes in line with values reported in literature [19,29,30], i.e., total cell counts were normal, and no inflammatory cells were present in the BAL cellular fraction (Figure 1). Regardless of the administration route, total BAL cell numbers did not increase in any of the amiodarone-treated groups tested (Figure 1). Neutrophils were elevated in a single animal in the day 1 cull group of the high-dose aerosol study (Protocol 2). However, as the animal numbers within this group were low and the effect transient, the significance of this outcome should not be overly interpreted. A significant (*p* < 0.001) increase in eosinophils was also observed in the high-dose aerosol group on cull day 7.

**Figure 2:**
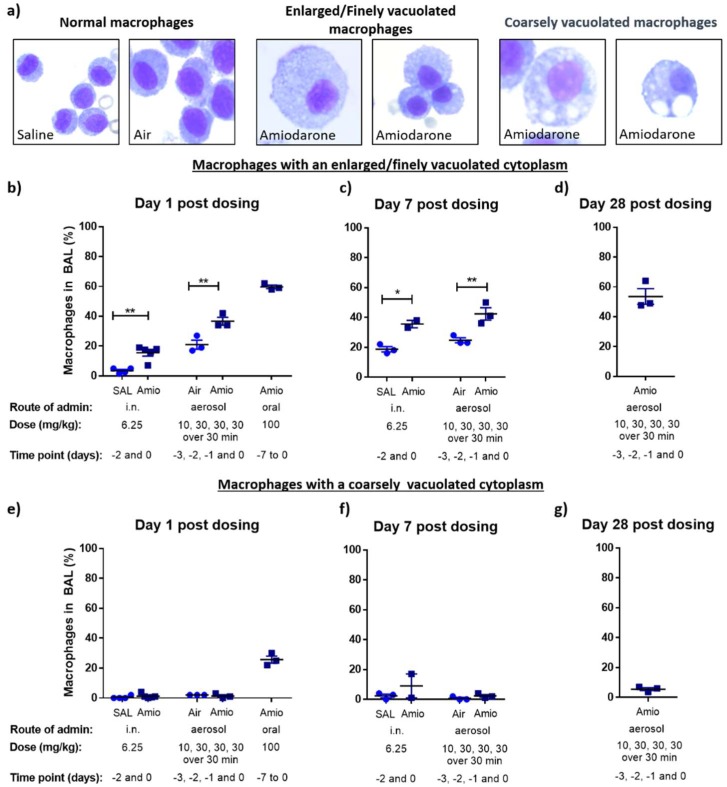
Morphological evaluation of BAL macrophages using light microscopy. (**a**) Representative images (63× magnification) showing macrophages from control animals (left image), enlarged macrophages with a finely vacuolated cytoplasm (centre image) and macrophages with a coarsely vacuolated cytoplasm (right image). The prevalence of macrophages with finely vacuolated cytoplasm (**b**–**d**) and coarsely vacuolated cytoplasm (**e**–**g**), expressed as a percentage of the total macrophage population at day 1, day 7 and day 28 post-dosing with vehicle control (saline (SAL) or air) and amiodarone. Data represents mean ± standard error of the mean (SEM) (*n* = 2–5 per treatment group); * *p* < 0.05, ** *p* < 0.01, statistically significant compared to respective control, two-way ANOVA with post hoc Bonferroni test, difference from untreated cells.

Light microscopy analysis of the BAL macrophage population revealed two distinct morphological phenotypes of vacuolated macrophages: enlarged macrophages with a finely vacuolated cytoplasm and macrophages with a coarsely vacuolated cytoplasm (Figure 2a). Previous morphological characterisation of amiodarone-treated macrophages from our group [4] and others [31,32] have observed that amiodarone exposure is associated with a characteristic vacuole morphology typified by one or two very large vacuoles, as shown in Figure 2a, while the enlarged, finely vacuolated phenotype may be associated with cells in the early stages of apoptosis [33]. In the current study, amiodarone treatment was associated with increased numbers of finely vacuolated macrophages for all administration routes, indicating a stress response in the macrophage population to the drug (Figure 2b–d). These numbers stayed high after dosing was terminated in the aerosol administration groups. In contrast, only the oral dosing protocol induced high numbers of the characteristic coarsely vacuolated cells (Figure 2e), typical following systemic amiodarone treatment.

**Figure 3:**
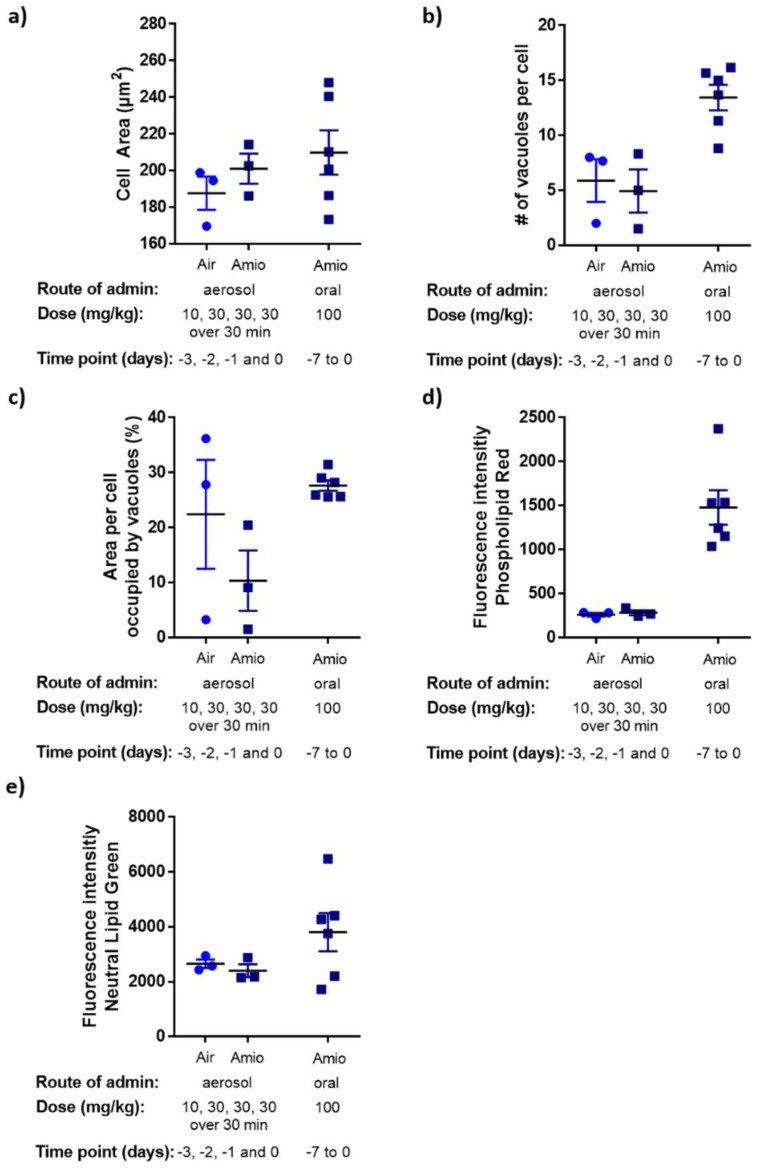
Median cell area (**a**), number of vacuoles per cell (**b**), percentage of cell area occupied by vacuoles (**c**), intensity of phospholipid stain (**d**) and neutral lipid stain (**e**) are reported for macrophages isolated from BAL on day 1 post-dosing. Each data point represents the mean ± standard error of the mean (SEM) of triplicate samples per rat (*n* = 3–6 animals). Data from day 7 of the aerosol studies did not show any elevation compared to controls and is reported in the supplementary material section (Figure S1).

The morphological evaluation using light microscopy was corroborated by the HCA assay of the isolated BAL macrophage population (Figure 3). Only the oral dosing group showed a consistently elevated percentage of the adherent macrophage population with a larger cellular area, a higher number of vacuoles, a larger vacuole area and, importantly, elevated phospholipid levels. These combined results indicate that oral treatment with high dose amiodarone promoted the development of phospholipidosis in AM without an accompanying lung inflammation, at least for time points immediately following treatment. The aerosol study, in contrast, failed to induce clear evidence of phospholipidosis in the BAL cellular fraction (Figure 3 and Figure S1).

### 3.2. Assessment of Inflammation and Phospholipidosis Markers in Lung Tissue

Lung histology from all groups showed only minor pathologies in a small number of animals, most of which were considered incidental (Table 1). Minor pathological observations (examples are depicted in Figure S2, Supplementary Materials section) were observed in the i.n. and both aerosol groups, while no significant pathology finding was observed in the oral dosing group. The minimal increases in alveolar macrophages observed here, especially in the i.n. and low-dose aerosol studies, would not be considered adverse in view of their low grades and absence of any associated degenerative findings. The observation of inflammatory cell infiltrates in the high-dose aerosol study could be an indication of a test-article related effect, since the occurrence is higher in the treatment groups compared to controls. It is relevant to note that the routine, blinded pathology evaluation of lung tissue slices did not yield conclusive reports of FAM observations for any treatment group, not even the oral dosing cohorts.

As a result of the discrepancies in outcomes between the models characterised here, especially between the aerosol and oral treatment regimens, it was hypothesised that both the administration route and dose are likely to have substantial effects on the amount and spatial distribution of drug reaching the lung. To assess this, MSI analysis was employed to characterise amiodarone distribution in the lung following oral and high-dose aerosol administration. The spatial distribution of the amiodarone metabolites, *n*-desyl-amiodarone, m8 and m11 [34] was evaluated, as well as the presence and distribution pattern of the phospholipidosis biomarker, di-22:6-bis-monoacylglycerol (BMP; [35]). However, the detection of BMP is complicated by the potential and even likely presence of di-22:6 phosphatidylglycerol (PG), a structural isotope of BMP.

**Figure 4:**
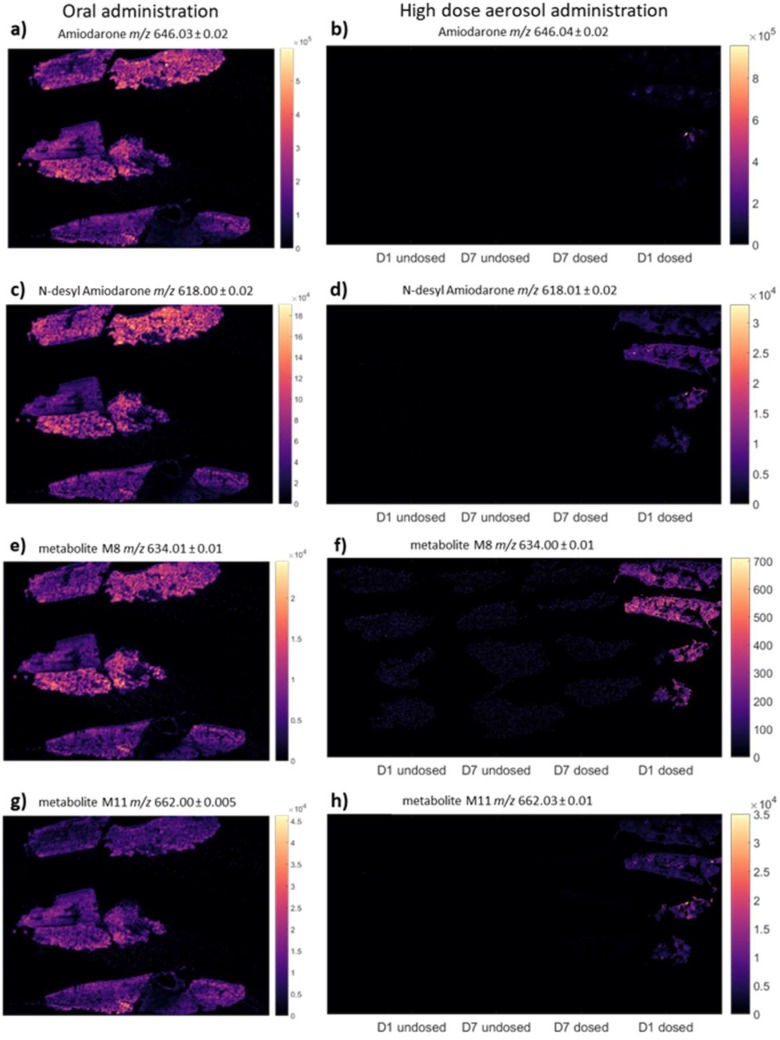
Selected ion images show the distribution of the major fragment ions of (**a**,**b**) amiodarone (*m/z* 646.03), (**c**,**d**) *n*-desyl amiodarone (*m/z* 618.00), and metabolites (**e**,**f**) M8 (*m/z* 662.02) and (**g**,**h**) M11 (633.99). Images A, C, E and G each represent 27.2 mm by 20.3 mm in the x and y dimension respectively. Images B, D, F and H each represent 46.5 mm by 23.3 mm in the x and y dimension respectively.

MSI analysis was able to detect amiodarone (total ion contribution of *m/z* 646.0214) in lung slices from animals on day 1 post-dosing in both oral and high-dose aerosol administration studies (Figure 4a,b). The spatial distribution of aerosolised amiodarone was typical for an inhaled compound with highly localised concentrations surrounding the airways denoting focal deposition, which contrasts greatly with the homogenous distribution of amiodarone following oral dosing. The three amiodarone metabolites, *n*-desyl-amiodarone, m8 and m11, were also predominantly present on day 1 only, showing a more homogenous distribution across lung tissue. Both amiodarone and its metabolites were no longer detectable one week post-aerosol dosing (Figure 4d,f,h).

**Figure 5:**
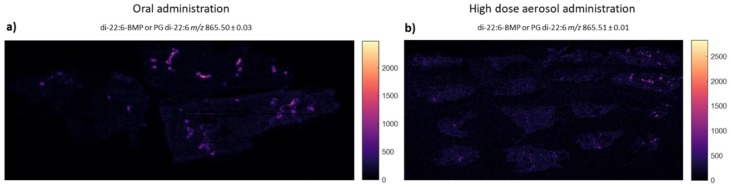
Ion images of *m/z* 865.51 in lungs of animals following oral (**a**) and aerosol (**b**) administration, showing a higher distribution of the target lipid in a highly localised area of the tissue (possibly major airways) along with a background in the tissue. Note: the signal could derive from the two isomeric species di-22:6 BMP and/or di-22:6 PG. Image A represents 44.3 mm by 17.4 mm in the x and y dimension respectively and image B 46.5 mm by 23.3 mm in the x and y dimension respectively.

Di-22:6-BMP is a surface-active lipid present in lung surfactant, as well as within lysosome and endosomes [36]. Elevated cellular concentrations of di-22:6-BMP have been increasingly associated with drug-induced phospholipidosis [35]. It was therefore of interest to determine whether elevated signals of di-22:6-BMP (*m/z* 865.50 with two characteristic fragments: *m/z* 283 and 327) were present in amiodarone-treated lung samples in this study. The images in Figure 5 show the distribution of the di-22:6-BMP parent ion, with a homogenously distributed, low-intensity signal present in all tissues (Figure 5). Notably, in both the day 1 cull groups from the oral and aerosol dosing studies, focal accumulations of di-22:6-BMP are present, often near airways. In the aerosol day 1 study group, the amiodarone and di-22:6-BMP signals are often co-localised.

## 4. Discussion

The accumulation of poorly soluble drugs has been identified as one cause of FAM responses [37], with some FAM responses being limited to aggregates at the bronchoalveolar junction; however, with others an associated neutrophilic inflammation is also observed [2]. Furthermore, the FAM distribution can vary depending on the route of administration. CADs administered systemically have FAMs distributed throughout the lungs, whereas, when CADs are inhaled FAMs are predominantly localised at the bronchoalveolar junction and the site of deposition [38].

Our study looked at a single strain of Wistar Han rats with amiodarone administered using four drug administration protocols. Overall, amiodarone administered at highly varying doses and by different routes (i.n., aerosol or oral gavage) was well tolerated, with only a mild inflammatory response observed in the high-dose aerosol study. A comparison of relevant model characteristics published across the literature highlights the variability observed between dosing protocols, administration routes and rat strains (Table 2). 

Two primary routes of administration are reported in the literature for amiodarone induced pulmonary toxicity (AIPT) in rats: oral and i.t. Oral dosing of animals leads to marked phospholipidosis in lung cells and tissue but does not produce a consistent pulmonary inflammatory response (Table 2). Conversely, i.t. dosing of animals produces an initial inflammatory response observed in BAL samples and lung tissue [18,19,20,39] subsequently followed by fibrosis [20,39,40]. A study comparing the pulmonary responses of hamsters to either oral or i.t. treatment concluded that oral amiodarone administration led to drug accumulation in lung tissue and significant phospholipidosis without resulting in fibrosis, whilst i.t. amiodarone treatment produced fibrosis without drug accumulation and phospholipidosis [12].

The reason for the discrepancy in response to amiodarone may be due to differences in amiodarone pharmacokinetics following different routes of administration. Amiodarone administered orally is absorbed slowly and incompletely, [11] and its metabolites have been reported to cause lung damage [15]. The parent compound, amiodarone, concentrates in organs with high lipid content (adipose tissues, thyroid and liver) and the lungs [41] with little detected in the blood [7]. Amiodarone also undergoes first pass metabolism by the enzyme cytochrome P450 in the liver and gastrointestinal tract, and only small amounts of amiodarone and its metabolites can be found in urine [11]. Amiodarone, once metabolised to its more polar counterpart *n*-desyl-amiodarone, accumulates in the lungs to a greater extent than the parent compound [8,9]. Thus, the more pronounced changes observed with amiodarone when administered by the oral route may be due to the ability of the lung to sequester amiodarone and in particular its metabolites [15]. This was supported in our study by MSI analysis of lung tissue showing evidence of *n*-desyl-amiodarone, m8 and m11, the two lesser known metabolites of amiodarone at day 1 post dosing in addition to the presence of the phospholipid marker, di-22:6-BMP. The HCA assay also indicated increased levels of phospholipid in BAL macrophages, a common observation following administration of amiodarone [7,8,17,42].

In contrast amiodarone exposure following inhalation does not undergo first pass metabolism [9] resulting in minimal lung damage due to the lack of production of toxic metabolites. The pulmonary inflammation observed following inhaled amiodarone may therefore be related to direct toxicity of the parent compound at higher doses, as observed with our high dose aerosol study, but not in the low dose aerosol study. However, the doses used in our inhalation study, which were limited by concerns over animal welfare and avoiding excessive lung inflammation, may have been too low to produce detectable changes in phospholipid content using the HCA assay, although a more sensitive method of analysis such as MSI was able to detect di-22:6-BMP in lung tissue slices. The distribution of the di-22:6-BMP marker following aerosolised administration was highly localised in the airways denoting focal deposition, which contrasts greatly with the homogenous distribution of amiodarone throughout lung tissue following oral dosing, and may limit the detection of phospholipids and allow for a more localised inflammatory response. There was no evidence of amiodarone or its metabolites at day 7 post dosing showing that amiodarone at the dose inhaled was not retained in the lungs and cleared more rapidly compared to chronic oral amiodarone administration.

In addition to differences observed following administration of amiodarone by different routes, there are considerable differences in AIPT according to the strain of rat used. A comparative study of orally treated Sprague-Dawley, Fischer 344 and Wistar Han rats revealed a marked sensitivity of Fischer 344 rats compared to the more moderate response of Sprague-Dawley rats (Table 2). A sub lethal dose of amiodarone induced lipid storage in the tissues of Fischer, and to a lesser extent Sprague-Dawley, rats, but not in Wistar rats [14]. This was further confirmed by Reasor et al. who reported that Sprague-Dawley and Long-Evans hooded rats were less susceptible to amiodarone induced pulmonary phospholipidosis than Fischer 344 rats [6]. Overall, Wistar Han rats in our study were relatively insensitive to AIPT under various experimental conditions and this has also been reported in previous studies [14,15], although, when administered at very high doses (>300 mg/kg) amiodarone has been shown to induce a mild toxic response [7]. The strain differences observed have been attributed to the high level of metabolite present in the lungs of Fischer 344 rats that are highly susceptible to the development of phospholipidosis and/or inflammation [15].

Despite the lack of pulmonary phospholipidosis observed in the Han-Wistar strain following aerosol administration, this investigation provided the opportunity to evaluate two relatively new analytical techniques, i.e., HCA and MSI, in the context of conventional inhalation toxicology studies. MSI, for example, provided vital information on the differences in drug localisation in the lung, thereby supporting interpretations regarding the relationship between drug distribution and foamy phenotype development. Further, MSI enabled detection and spatial mapping of the putative phosopholipidosis biomarker, di-22:6-BMP, on a tissue level, which could potentially be used in toxicological assessments to complement BAL analysis.

The fluorescence-based HCA methodology is currently used predominantly in an in vitro setting to screen large numbers of compounds for cytotoxicity, morphological features and lipid profiling [4,24] prior to animal studies. To examine whether the in vitro culture conditions used in HCA screens reflect macrophage responses in vivo, it is important to analyse cells harvested from an in vivo exposure model and directly compare with in vitro data. We previously reported in vitro morphometric data and neutral lipid/phospholipid content in the rat-derived NR8383 cell line, human-derived, differentiated U937 cells and rat primary alveolar macrophages harvested from the BAL of naïve Han Wistar rats [24]. The cell lines and primary cells were cultured under submersed conditions and exposed to 10 µM amiodarone for 24 and 48 h. Direct comparison of the 48 h ex vivo exposure data with the HCA analysis of BAL macrophages harvested from rats receiving oral amiodarone treatment for seven days (Figure 3) revealed similar trends in the macrophage responses (Table 3), although all measured parameters were slightly elevated in cells from the orally dosed animals (Table 3). This elevated response may result from a longer exposure time and higher exposure concentrations to amiodarone achieved in vivo compared to the in vitro model. Nonetheless, the data justify the use of the rat cell line, NR8383, in in vitro screening assays, since the results are comparable to cultured primary cells from the same species. Further studies will focus on the comparison of human-derived macrophage cell lines and primary human alveolar cells harvested from lavage, as well as species differences.

## 5. Conclusions

The current study was used to investigate two main research questions: first, does aerosol administration of amiodarone provide a better model of pulmonary FAM for safety investigations of inhaled medicines? Secondly, do FAM harvested from the BAL of amiodarone-treated rats show similar characteristics to cultured macrophage cells treated in vitro with amiodarone? Our present findings indicate that inhaled amiodarone induces transient pulmonary inflammation with little damage to the lungs of Wistar rats, and induces some finely vacuolated AM with no change in macrophage numbers. However, the prevalence of FAM was not as prominent as has been reported following systemic administration of this drug. These observations suggest that the FAM-inducing effect of amiodarone is best achieved by systemic administration and that inhaled amiodarone cannot be used as a positive control for inducing FAM in studies investigating the safety of inhaled medicines in vivo. Nonetheless, the study did reveal that macrophages harvested from the BAL of rats dosed orally with amiodarone over seven days showed similar trends in a fluorescence-based HCA assay, indicating that in vitro testing of novel compounds for FAM-inducing effects may be predictive of FAM development in vivo.

## Figures and Tables

**Table 1:** Summary of pathology observations in study groups with an observed pathology. Groups with no pathology observations are not listed. Results depict the number of animals per cohort exhibiting the pathology.

Cohort Description			Pathology		
Dosing Method	Treatment	Cull Day	Minimal Increases in Alveolar Macrophage Numbers	Foci of Foamy Cells	Minimal Perivascular/Peribronchiolar Inflammatory Cell Infiltrate (Consisting of Neutrophils and Eosinophils)
i.n.	Control	1	2/2	0/2	0/2
	Amiodarone	1	2/2	0/2	0/2
	Control	7	3/3	0/3	0/3
	Amiodarone	7	3/3	0/3	0/3
Aerosol: low dose	Control	1	0/6	0/6	0/6
	Amiodarone	1	3/6	1/6	0/6
	Control	7	3/6	0/6	0/6
	Amiodarone	7	3/6	0/6	0/6
	Control	28	2/6	0/6	0/6
	Amiodarone	28	3/6	0/6	0/6
Aerosol: high dose	Control	1	0/6	0/6	0/6
	Amiodarone	1	2/6	0/6	3/6
	Control	7	1/6	0/6	1/6
	Amiodarone	7	1/6	0/6	6/6

**Table 2:** Overview of the characteristics of amiodarone induced phospholipidosis models.

Study Information				Reported Outcomes					
Route	Rat Strain	Amiodarone Dosing Protocol	Authors	Total BAL Counts (×10^6^ cells/mL)	BAL Neutrophils (×10^6^ cells/mL)	BAL Eosinophils (×10^6^ Cells/mL)	Macrophage Phospholipid Accumulation	Lamellar Bodies	Foci of Foamy Cells in Tissue
Oral	HW ^1^	Rats fed 175, 300, 400 or 500 mg/kg for 6 wk suspended in 0.5% methyl cellulose for 5 days/wk by gavage	Wilson et al., 1991 [7]	High dose group had significantly more cells than control or the 175 mg/kg group.	175 mg/kg < 0.5 300 mg/kg < 2 400 mg/kg ~ 2 500 mg/kg ~ 3	Not reported	Yes (Only in the three high dose groups)	No	Yes (Only in the three high dose groups)
	F344 ^2^	Rats treated orally (50, 100, 150 or 200 mg/kg), daily for 1 wk (5 days/wk)	Reasor et al., 1988 [6]	Not reported	Not reported	Not reported	Yes (Only in the two high dose groups)	Yes	Yes
	LE ^3^	Rats treated orally 150 mg/kg, daily for 1 wk (5 days/wk)	Reasor et al., 1988 [6]	N/A	Not reported	Not reported	No	N/A	N/A
	SD ^4^	Rats treated orally 150 mg/kg, daily for 1 wk (5 days/wk)	Reasor et al., 1988 [6]	N/A	N/A	N/A	No	N/A	N/A
	HW	Rats fed 175 mg/kg suspended in 0.5% methyl cellulose 5 days/wk by gavage	Wilson and Lippmann, 1990 [15]	No increase in BAL cell counts	No	Not reported	N/A	N/A	Some intra-alveolar FAM
	F344	Rats fed 175 mg/kg suspended in 0.5% methylcellulose 5 days/wk by gavage	Wilson and Lippmann, 1990 [15]	Total lavage cell count was increased (*p* < 0.001)	Increased neutrophils (*p* < 0.001)	Not reported	N/A	N/A	AM ^5^ were much larger than the HW AM. Alveolar spaces contained large FAM ^6^
	F344	Fed 150 mg/kg for 2 weeks by gavage	Mazue et al., 1984 [14]	N/A	N/A	N/A	Yes (Lymph nodes and lungs)	Yes	Yes, distended FAM with pale, finely vacuolated cytoplasm
	HW	Fed 150 mg/kg for 2 weeks by gavage	Mazue et al., 1984 [14]	N/A	N/A	N/A	No	N/A	N/A
	SD	Fed 150 mg/kg for 2 weeks by gavage	Mazue et al., 1984 [14]	N/A	N/A	N/A	Yes (Lymph nodes only)	N/A	N/A
	HW	100 mg/kg suspended in 1% *w*/*v* methyl cellulose for seven consecutive days	Patel et al., 2019	1.36	0.00	0.01	Yes (slight increase)	N/A	No
i.n.	HW	6.25 mg/kg, dosed twice at days −2 and 0	Patel et al., 2019	0.70	0.04	0.03	No	N/A	No
i.t.	F344	6.25 mg/kg, dosed twice at days 1 and 3	Taylor et al., 2000 [18]	~56	~14	~1.4	N/A	No	Not reported
	F344	6.25 mg/kg, dosed twice at days 1 and 3	Lee et al., 2013 [19]	~5	~2	~0.8	Yes (Formation of lipid droplets)	Yes	No
Aero.	HW	~3 mg/kg over 30 min on day −2 and 0	Patel et al., 2019	0.91	0.01	0.00	No	N/A	No
	HW	10 mg/kg (day −3) and 30 mg/kg (days −2, 1 and 0)	Patel et al., 2019	1.84	0.06	0.03–0.07	No	N/A	No

^1^ HW = Han Wistar, ^2^ F344 = Fischer 344, ^3^ LE = Long-Evans, ^4^ SD = Sprague-Dawley; ^5^ AM = alveolar macrophages, ^6^ FAM = Foamy Alveolar Macrophages.

**Table 3:** Comparison of morphometric parameters and neutral lipid/phospholipid content between the rat cell line, NR8383, primary rat BAL macrophages from naïve rats exposed ex vivo to 10 µM amiodarone [24] and primary rat BAL macrophages harvested from Han Wistar rats treated orally with amiodarone (Figure 3).

Parameter	Rat NR8383 Cell Line [24]	Rat Primary BAL Macrophages: Ex Vivo 48 h Exposure [24]	Rat Primary BAL Macrophages: Oral Dosing Study
Cell area (span of values) (µm^2^)	113–201	165–265	170–250
Median number of vacuoles per cell	5	10	14
Median vacuole area per cell area (%)	10	12	27
LipidTOX^TM^ red fluorescence intensity fold-change: amiodarone vs. untreated/naive	2.7	2.8	6.0
LipidTOX^TM^ green fluorescence intensity fold-change: amiodarone vs. untreated/naive	1.3	1.2	1.6

## Supplementary Materials

The following are available online at https://www.mdpi.com/1999-4923/11/7/345/s1, Figure S1: Median cell area (**a**), number of vacuoles per cell (**b**), percentage of cell area occupied by vacuoles (**c**), intensity of phospholipid stain (**d**) and neutral lipid stain (**e**) are reported for macrophages isolated from BAL on day 7 post-dosing. Each data point represents the mean ± standard error of the mean (SEM) of triplicate samples per rat (*n* = 3–6 animals), Figure S2: Histopathology (×10 magnification) of lung tissue exposed to amiodarone. Representative images of lung tissue harvested at day 1 and day 7 following treatment (amiodarone) or air/saline control.
