# Supplementary material for: Comparison of Oral, Intranasal and Aerosol Administration of Amiodarone in Rats as a Model of Pulmonary Phospholipidosis
Source: Pharmaceutics. 2019 Jul 17;11(7):345. doi: 10.3390/pharmaceutics11070345 (PMC6680908; doi:10.3390/pharmaceutics11070345)
Supplement: Supplementary file 1 [file pharmaceutics-11-00345-s001.pdf]

*pharmaceutics*

MDPI

# Supplementary Materials: Comparison of Oral, Intranasal and Aerosol Administration of Amiodarone in Rats as a Model of Pulmonary Phospholipidosis

**Aateka Patel, Ewelina Hoffman, Doug Ball, Jan Klapwijk, Rory T. Steven, Alex Dexter, Josephine Bunch, Daniel Baker, Darragh Murnane, Victoria Hutter, Clive Page, Lea Ann Dailey and Ben Forbes**

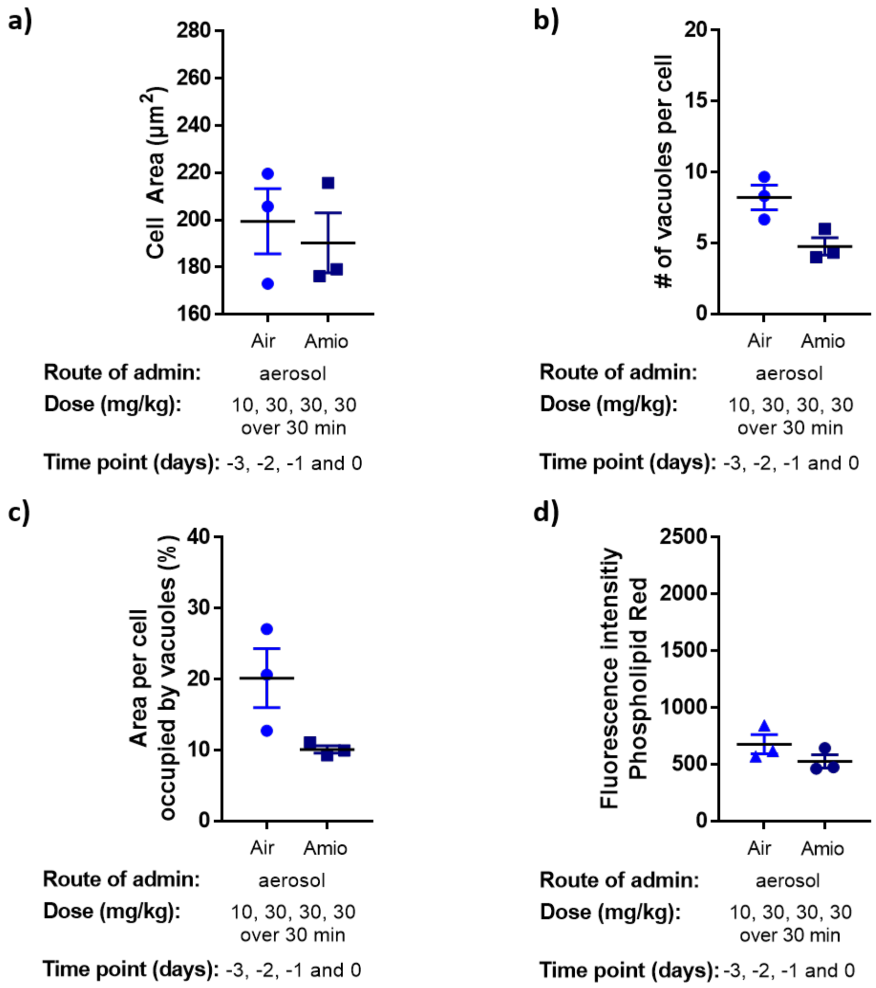

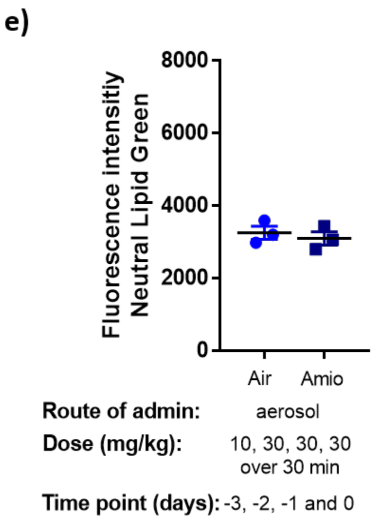


**Figure S1.** Median cell area (**a**), number of vacuoles per cell (**b**), percentage of cell area occupied by vacuoles (**c**), intensity of phospholipid stain (**d**) and neutral lipid stain (**e**) are reported for macrophages isolated from BAL on day 7 post-dosing. Each data point represents the mean ± standard error of the mean (SEM) of triplicate samples per rat ($n$ = 3–6 animals).

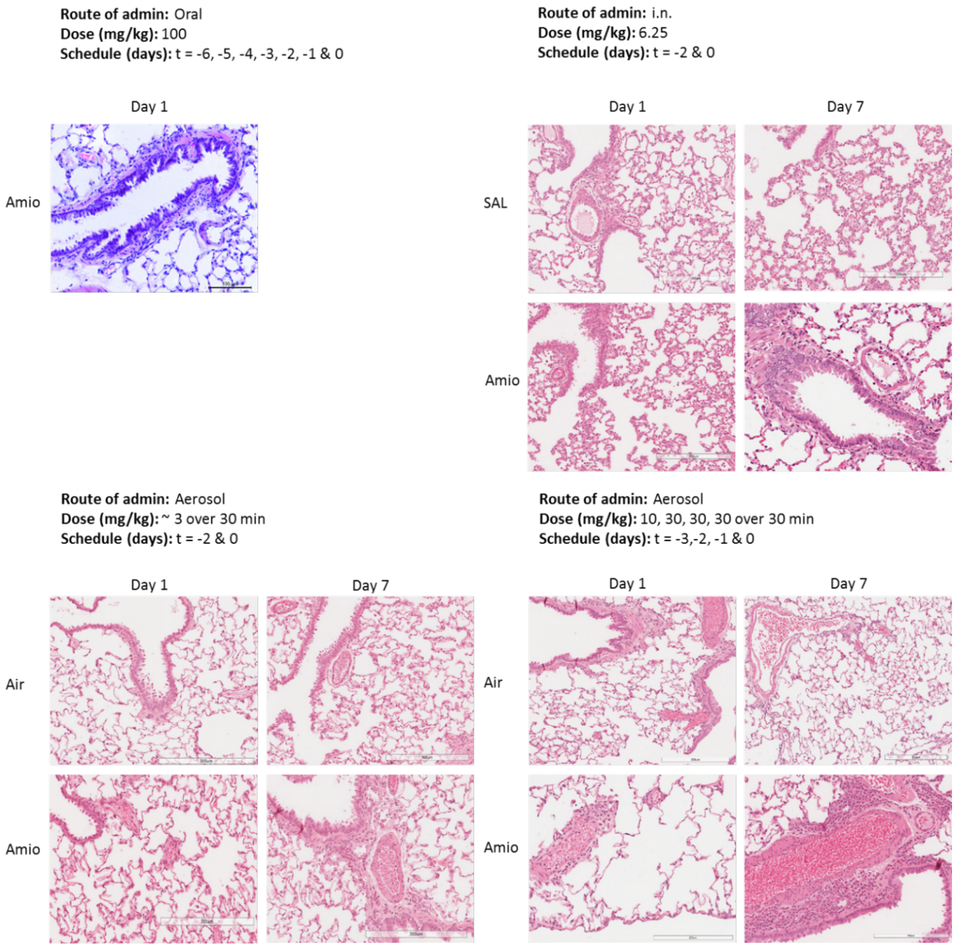


**Figure S2.** Histopathology (×10 magnification) of lung tissue exposed to amiodarone. Representative images of lung tissue harvested at day 1 and day 7 following treatment (amiodarone) or air/saline control.
